# Lack of molecular mimicry between HPV vaccine L1 antigen and human proteins by a computational analysis

**DOI:** 10.1007/s10147-026-02961-z

**Published:** 2026-01-08

**Authors:** Kazuhiro Nishioka, Kentaro Sekiyama, Reona Shiro, Ikuo Tsunoda, Noriomi Matsumura

**Affiliations:** 1 Department of Obstetrics and Gynecology, Kindai University Nara Hospital, 1248-1, Otodacho, Ikoma, Nara 630-0293, Japan; 2 Department of Microbiology, Faculty of Medicine, Kindai University, 1-14-1, Miharadai, Minami-ku, Sakai, Osaka 590-0197 Japan; 3 Department of Obstetrics and Gynecology, Faculty of Medicine, Kindai University, 1-14-1, Miharadai, Minami-ku, Sakai, Osaka 589-8511 Japan

**Keywords:** Amino Acid Sequences, Autoimmunity, Cross Reactions, HANS, Uterine Cervical Neoplasms

## Abstract

**Background:**

Although human papillomavirus (HPV) vaccines effectively prevent cervical cancer, the HPV vaccination rates in Japan remain low because of concerns about alleged neurological adverse events. Darja Kanduc proposed a flawed hypothesis that molecular mimicry between HPV and human proteins could induce cross-reactive antibodies, causing autoimmune organ damage, even when only the portions of amino acid (AA)-sequences of the epitopes were identical between HPV and human proteins.

**Methods:**

In this study, we conducted the same computational data analysis as Kanduc, using 22 linear epitopes (9–23 AA-length) of the HPV type 16 L1 protein (HPV16L1) registered in the database.

**Results:**

We found that no human epitopes had identical AA-sequences to any HPV16L1 epitopes, demonstrating that HPV16L1 had no molecular mimicry with linear epitopes that have the potential to induce cross-reactive autoantibodies. On the other hand, we identified various numbers of human protein epitopes whose AA-sequences were partially identical with epitopes of HPV16L1, hepatitis B virus (HBV), and respiratory syncytial virus (RSV). We found that HPV16L1 had a smaller number of such proteins having “partial molecular mimicry” than HBV and RSV.

**Conclusions:**

Our current in silico analysis provided no evidence that HPV vaccinations could induce cross-reactive autoantibodies. The flawed molecular mimicry data should not be used as a scientific basis for alleged HPV vaccine-induced adverse events.

**Supplementary Information:**

The online version contains supplementary material available at 10.1007/s10147-026-02961-z.

## Introduction

Cervical cancer is the fourth most common malignant neoplasm worldwide. In Japan, about 10,000 people were diagnosed with cervical cancer, among which 3000 people died, annually [1]. Persistent human papillomavirus (HPV) infection is the major cause of cervical cancer, of which HPV type 16 (HPV16) and HPV18 accounted for about 70% [2]. HPV vaccines contain a recombinant protein of the HPV L1 protein and an adjuvant. Bivalent HPV vaccines (2vHPV) have been developed for the prevention of HPV16 and HPV18 infections. A quadrivalent vaccine (4vHPV) can prevent infections caused by HPV6, HPV11, HPV16, and HPV18. Both 2vHPV and 4vHPV can prevent nearly 100% of HPV16 and HPV18 infections, as well as almost all precancerous lesions caused by HPV16 and HPV18 in uninfected individuals [3, 4]. Recently, it has been reported that the efficacy of the nonavalent HPV vaccine (9vHPV) in preventing infections with HPV31, 33, 45, 52, and 58, in addition to the four HPV types included in the 4vHPV [5]. The World Health Organization has concluded that all three HPV vaccines (2vHPV, 4vHPV, and 9vHPV) has an excellent safety and efficacy profile and recommended that HPV vaccines be included in national immunization programs [6].

In Japan, HPV vaccinations began in 2010 and became routine in April 2013. In June 2013, however, widespread media coverage of "diverse symptoms," or a novel disease entity, “HPV vaccination-associated neuro-immunopathic syndrome (HANS),” characterized by widespread pain and neuro-psychological abnormalities, following HPV vaccinations, led to a suspension of proactive vaccination recommendations. The HPV vaccination coverage rate became less than 1% [7]. Later, national and international studies denied the causal relationship between HPV vaccinations and the "diverse symptoms" [8]. Although the proactive recommendations of HPV vaccinations resumed in April 2022, the HPV vaccination rate in Japan has remained low [9].

Darja Kanduc argued that, since portions of amino acid (AA) sequences of HPV and human proteins are highly homologous, HPV vaccinations might induce autoantibodies that could cause organ damage, leading to autoimmune diseases and multiple sclerosis (MS) [10–12]. Clinically and experimentally, however, no studies have demonstrated the presence of such cross-reactive autoantibodies in human or animal sera following HPV vaccinations. In addition, increases in autoimmune diseases and any other diseases following HPV vaccinations have been denied, epidemiologically [13].

Kanduc compared the AA-sequences between all proteins of HPV16 (structural L1 and L2 and non-structural E1-E7 proteins) versus the human proteome. Kanduc claimed that large numbers of 7AA- or 8AA-length sequences, which were designated as “heptapeptide (or heptamer, 7-mer motif)” or “octapeptide,” respectively [14], and 5AA- or 6AA-length sequences (“pentapeptide, 5-mer motif” or “hexapeptide, 6-mer motif”) [15] were commonly present in HPV and human proteins. Kanduc’s data cannot be used as evidence that the HPV vaccine causes autoantibodies, since her manuscripts had the following scientific flaws. Although Kanduc compared AA-sequences of all HPV proteins with human proteins in her first report [14], the HPV vaccines contain only the HPV L1 protein, not the other HPV proteins (L2 and E1-E7). In addition, antibodies recognize and bind to specific AA-sequences of the antigen, which is called an epitope. Although only the molecular mimicry between HPV L1 epitopes and host protein epitopes has the potential cross-reactive response, Kanduc compared the entire AA-sequences of HPV proteins and human proteomes, including non-epitope regions [15]. Later, Kanduc et al. compared a portion of the linear epitopes of HPV16 L1 protein (HPV16L1) with those of human proteins based on the erroneous hypothesis that if a partial sequence of the epitope matched a human protein, it could produce autoantibodies [16]. Immunologically, however, since epitopes can be defined as the minimum antigenic structures recognized by antibodies, the discoveries of the similarities between portions of microbial epitopes and human proteins, instead of the full-length AA-sequence of them, were irrelevant to cross-reactivities of anti-microbial antibodies to human proteins. Despite the scientific flaws in Kanduc's computational analyses, no group has used the same methodology to test the reproducibility of molecular mimicry between HPV and host proteins published by Kanduc. Consequently, Kanduc’s “discoveries” of the molecular mimicries have been cited as "scientific evidence" in some scientific manuscripts [15] and in lawsuits by anti-vaccine activists.

In this study, we performed a computational analysis using the same methodology as Kanduc’s group. This study will help clarify the understanding of HPV vaccines by showing that the claim that HPV vaccines induce autoantibodies through molecular mimicry is incorrect.

## Materials and methods

### Molecular mimicry analysis between the HPV16L1 and human epitopes

Using the method described by Kanduc et al. [16], we identified the linear epitopes of the B cell receptor (antibody) against the HPV16 L1 in the Immune Epitope Database (IEDB) (https://www.iedb.org/). In the IEDB, no L1 epitope of HPV type 18 was registered. Then, we compared the AA-sequences of HPV16 L1 epitopes of the human antibodies and the human protein epitopes of the human antibodies. The Basic Local Alignment Search Tool (BLAST) (https://blast.ncbi.nlm.nih.gov/Blast.cgi?PAGE=Proteins) was used to determine whether the same AA-sequences in the HPV16 L1 epitopes could be found in human antibody epitopes of human proteins as described previously by Kanduc et al. [15]. The AA-sequence of interest was entered into the Query Sequence field. We selected “RefSeq Select proteins” for the Database in Search Set field. We entered “Homo sapiens” for the Organism. We used protein–protein BLAST (blastp) to search human protein epitopes whose sequences were identical to the AA-sequences of interest (viral sequences or random array sets) with algorithm parameters: Percent Identity (Per Ident.), 100%; and Query Cover, 100%. We examined not only the AA-sequences of the full length of the linear epitopes, but also the portions of AA-sequences of the epitopes from five AA-length (pentapeptide) to 27 AA-length [15].

### Molecular mimicry analysis using HBV and RSV

We identified the linear epitopes of the full-length hepatitis B virus (HBV) [17] and respiratory syncytial virus (RSV) [18] on human B cell receptors in the IEDB. From the sequences of these HBV and RSV epitopes, we randomly generated five sets of the same numbers of AA-sequences as those generated from the HPV L1 epitopes: HBV sets 1–3 and RSV sets 1 and 2 (Tables S1-S5, note: in RSV set 2, we were not able to make the same numbers, but smaller numbers of 10AA-peptide, 11AA-peptide, 12AA-peptide and 13AA-peptide). Then, using the BLAST, we determined the numbers of AA-sequences among the HBV and RSV epitopes, which were identical to those of human B cell epitopes. HBV epitopes were from four open reading frames (ORFs): large hepatitis B surface antigen (PreS1, PreS2, and HBs); pre-core/core ORF (HBc and HBe); HBx (protein X); and DNA polymerase [19]. RSV epitopes were from F protein, G protein, N protein, and P protein [20].

### Molecular mimicry analysis using random AA-sequences and gene ontology analysis

We generated four sets of random AA-sequences (Random array sets 1–4, Tables S6-S9), which contained the same AA-length and sequence numbers as those of HPV16L1, using Excel's random number generator. We used the BLAST to identify the AA-sequences that matched human protein epitopes. We also conducted gene ontology analysis using the list of identified human proteins that shared the AA-sequences with AA-sequences of Random array sets, using the Gene Ontology Resource (https://geneontology.org/).

### Statistical analysis

Welch's *t*-test was used to compare the number of human proteins sharing the same AA-sequences with virus-derived and random sequences. Statistical analysis was performed using Prism 10.4 (GraphPad Software, Boston, MA).

## Results

### AA-sequence comparison between HPV and human epitopes

To analyze molecular mimicry between HPV L1 and human proteins, we used 22 linear epitopes of HPV16L1, whose AA-sequence lengths ranged from 9 to 27, registered in the IEDB (Table 1). We found that no human proteins had identical sequences to the entire AA-sequences of the 22 linear epitopes. Thus, this computational analysis provided evidence that no antibodies against any epitopes of HPV16L1 would function as autoantibodies against human proteins.

**Table 1:** Linear epitopes of HPV16 L1 protein

HPV epitope ID	Epitope	AA length	5-peptide	6-peptide	7-peptide	8-peptide	9-peptide	10-peptide	11-peptide	12-peptide	13-peptide	References and DOI
109,332	IHSMNSTIL	9	5	4	3	2	1	-	-	-	-	Zavaleta R et al. *J Gen Virol* 2004 10.1099/vir.0.80077–0
110,602	GLKAKPKFTLGKRKATPTT	19	15	14	13	12	11	10	9	8	7	Le Cann P et al. *J Med Virol* 1995 10.1002/jmv.1890450410
110,651	LCLIGCKPPIGEHWGKGSP	19	15	14	13	12	11	10	9	8	7	Lenner P et al. *Cancer Immunol* 1995 10.1007/BF01517352
110,652	LCLIGCKPPIGEHWGKGSPC	20	16	15	14	13	12	11	10	9	8	Dillner J et al. *Int J Cancer.* 1990 10.1002/ijc.2910450326
110,733	VGENVPDDLYIKGSG	15	11	10	9	8	7	6	5	4	3	Le Cann P et al. *J Med Virol* 1995 10.1002/jmv.1890450410
110,825	EDTYRFVTSQAIACQKHTPPA	21	17	16	15	14	13	12	11	10	9	Leon S et al. *Sex Transm Dis.* 2009 10.1097/OLQ.0b013e318195762c
110,863	GLKAKPKFTLGKRKATPTTS	20	16	15	14	13	12	11	10	9	8	Cason J et al. *Int J Cancer.* 1992 10.1002/ijc.2910500304
110,872	GSGSTANLASSNYFP	15	11	10	9	8	7	6	5	4	3	
110,898	IACQKHTPPAPKEDPLKKYTFWEVNLK	27	23	22	21	20	19	18	17	16	15	Sharma BK et al. *Eur J Cancer.* 1996 10.1016/0959–8049(96)00005–6
110,938	LYIKGSGSTANLASSNYFPT	20	16	15	14	13	12	11	10	9	8	Leon S et al. *Sex Transm Dis.* 2009 10.1097/OLQ.0b013e318195762c
110,965	PNNNKILVPKVSGLQYRVFR	20	16	15	14	13	12	11	10	9	8	
111,088	ACQKHTPPAPKEDDPLKKYT	20	16	15	14	13	12	11	10	9	8	Dillner J et al. *Int J Cancer.* 1990 10.1002/ijc.2910450326
111,450	KGSPCTNVAVNPGDCPPLEL	20	16	15	14	13	12	11	10	9	8	
111,581	NGICWGNQLFVTVVDTTRST	20	16	15	14	13	12	11	10	9	8	
111,583	NKFGFPDTSFYNPDTQRLVW	20	16	15	14	13	12	11	10	9	8	
111,585	NKSEVPLDICTSICKYPDYI	20	16	15	14	13	12	11	10	9	8	
111,890	VDNRECISMDYKQTQLCLIG	20	16	15	14	13	12	11	10	9	8	
111,915	VHTGFGAMDFTTLQANKSEV	20	16	15	14	13	12	11	10	9	8	
175,631	PLGVGISGHPLLNKLDDTEN	20	16	15	14	13	12	11	10	9	8	Yokomine M et al. *Exp Ther Med.* 2017 10.3892/etm.2017.4150
175,636	PTPSGSMVTSDAQIFNKPYW	20	16	15	14	13	12	11	10	9	8	
175,665	VTSDAQIFNKPYWLQRAQGH	20	16	15	14	13	12	11	10	9	8	
604,717	TSDAQIFNKP	10	6	5	4	3	2	1	-	-	-	
Total sequence number			327	305	283	261	239	217	196	176	156	
Subtracted total sequence number^*^ = Total sequence number – overlapped sequence number			248	237	225	211	196	181	167	152	137	

AA, amino acid; DOI, digital object identifier

^*^Subtracted total sequence number (= the precise total sequence number) was determined by subtracting the number of overlapped AA sequences among all AA sequences from the total sequence number. For example, the 3rd ID 110651 and the 4th ID 110652 were almost identical except for the 3rd ID had only one additional AA; 19 AA sequences were overlapped in two IDs. The 10-AA length epitope of the last ID 604717 was included in the two preceding ID 175636 and ID 175665. Subtracting these duplicate sequences yields the precise total number

Next, using the same approach as described by Kanduc [15], we examined the number of human proteins that share the portion of the AA-sequences of the HPV16L1 epitopes, although sharing the portion of AA-sequences of the HPV16L1 epitopes with human protein epitopes alone does not result in the production of cross-reactive autoantibodies. For example, when we analyzed an AA-sequence identity of one 9-mer HPV16L1 epitope, IHSMNSTIL (Epitope ID 109332, Table 1), to the human epitopes at the consecutive seven AA-sequence level, which was called “heptapeptide,” “heptamer,” or “7-mer motif” by Kanduc [14], one can identify three heptapeptides, each peptide of which was offset by one residue, overlapping by six residues: i.e., 1) IHSMNST, 2) HSMNSTI, and 3) SMNSTIL. Using all the AA-sequences of the 22 linear epitopes, consisting of 9–27 AA-sequences, we were able to generate 248 pentapeptides (5AA-length). Then, using the BLAST search, we found that 1281 human protein epitopes shared at least one of these 248 pentapeptides (Table 2). Using the same method, we generated 237 hexapeptides (6AA-length) from the AA-sequences of the 22 epitopes; 106 human proteins shared at least one of these hexapeptides (Table 2). Similarly, 225 heptapeptides (7AA-length) were generated from the 22 epitopes shared with 16 human proteins; 211 octapeptides (8AA-length) shared with two human proteins; 196 nonapeptides (9AA-length) shared with two human proteins. No human protein had a sequence that was identical to the AA-sequence of HPV16L1 in 10AA or more than 10AA-length (Table 2).

**Table 2:** Number of human proteins with AA-sequences identical to viral epitope AA-sequences

Virus		Viral protein	AA length (number of AA sequences)								
			5-peptide	6-peptide	7-peptide	8-peptide	9-peptide	10-peptide	11-peptide	12-peptide	13-peptide
			(248)	(237)	(225)	(211)	(196)	(181)	(167)	(152)	(137)
HPV		HPV16 L1	1281	106	16	2	2	0	0	0	0
HBV	Set 1	Large hepatitis B surface antigen	1695	127	16	5	3	0	0	0	0
		core protein									
		HBx									
	Set 2	Large hepatitis B surface antigen	1549	104	17	5	4	1	0	0	0
		core protein									
		HBx									
	Set 3	Large hepatitis B surface antigen	1667	167	27	16	9	0	0	0	0
		core protein									
		HBx									
		DNA polymerase									
RSV	Set 1	G protein	1491	108	19	6	4	1	0	0	0
		N protein									
		F protein									
	Set2	G protein	1720	117	24	12	7	4	1	1	0
		N protein									
		F protein									
		P protein									
Random array	Set 1	Not applicable	825	50	8	5	1	0	0	0	0
	Set 2		972	60	13	4	3	1	0	0	0
	Set 3		578	28	2	1	0	0	0	0	0
	Set 4		649	39	10	2	0	0	0	0	0

AA, amino acid; HBV, hepatitis B virus; HPV, human papillomavirus; RSV, respiratory syncytial virus

### HBV and RSV epitopes had larger numbers of AA-sequence homology with human epitopes than HPV epitopes

There were 249 linear epitopes registered in the IEDB that used the full-length HBV protein as an antigen. From these, we randomly selected epitopes consisting of 9–27 AAs. From these epitopes, we generated the same number of AA-sequences as from the HPV16L1 epitopes, namely 248 pentapeptides (5AA-length); 237 hexapeptides (6AA-length); heptapeptides (7AA-length); 211 octapeptides (8AA-length); 196 nonapeptides (9AA-length); 181 10AA-peptides; 167 11AA-peptides; 176 12AA-peptides; and 138 13AA-peptides. Then, we generated three sets of these AA-sequence groups (HBV sets 1, 2, and 3) and examined the number of human proteins sharing these AA-sequences in HBV sets 1, 2, and 3 (Table 2**, **Tables S1-S3). Similarly, 159 linear epitopes were registered in the IEDB for the full-length human RSV protein. We followed the same approach for RSV as we did for HBV, creating two sets of groups of AA-sequences (RSV sets 1 and 2) and determining the number of human proteins that contained these AA-sequences (Table 2, Tables S4-S5). We compared the shared AA-sequence numbers at 5AA-, 6AA-, 7AA-, 8AA-, 9AA-, and 10AA-length levels. We found that the shared AA-sequence numbers in HPV epitopes were lower than those of HBV and RSV epitopes at all AA-length levels.

### Viral epitopes had more shared AA-sequences with human epitopes than random AA-sequences

**Fig. 1:**
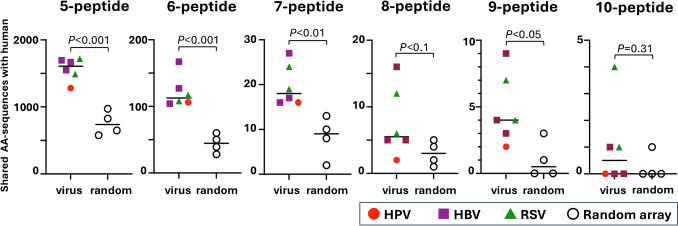
Comparison of the numbers of human amino acid (AA) sequences identical to viral AA-sequences or random array sequences at 5-peptide (pentapeptide), 6-peptide (hexapeptide), 7-peptide (heptapeptide), 8-peptide (octapeptide), 9-peptide (nonapeptide), or 10-peptide levels. We determined the shared AA-sequence numbers using the epitope data of human papillomavirus (HPV) 16 L1 (closed circle), hepatitis B virus (HBV) sets 1 to 3 (closed square), respiratory syncytial virus (RSV) sets 1 and 2 (closed triangle), and Random array sets 1 to 4 (open circle). At all AA-length levels, the shared AA-sequence numbers in HPV epitopes were lower than those of HBV and RSV epitopes. Welch's *t*-test was used to compare the numbers of human protein epitopes sharing the same AA-sequences with combined virus epitopes (HPV, HBV, and RSV) versus Random array sets 1 to 4.

We also randomly generated 9–27 AA-length sequences, from which we generated four sets of 5–13 AA-sequences (Random array sets 1, 2, 3, and 4) to determine the numbers of human epitope sequences identical to these random AA-sequences (Table 2**, Tables S6-S9**). When comparing the number of matching human epitopes for the three viral epitopes (HPV, HBV, and RSV combined) and for the Random array sets, the former were more common for penta-, hexa-, hepta-, octa- and nonapeptides (5 to 9 AA-length peptides). For example, at pentapeptide (5AA-length peptide) level, HPV had 1281; HBV sets 1–3 had 1549 to 1695; RSV sets 1–2 had 1491 to 1720; and Random array sets 1–4 had 825 to 972 pentapeptides commonly found in human protein epitopes (Table 2, Fig. 1). Statistically, the mean numbers of the common AA-sequences ± standard error of the mean (SEM) were as follows (Fig. 1): 5AA-length, viral epitopes 1567.2 ± 67.7, random array, 756.0 ± 88.8, *P* < 0.001; 6AA-length, viral epitopes 121.5 ± 9.7, random array 44.3 ± 6.9, *P* < 0.001; 7AA-length, viral epitopes 19.8 ± 1.9, random array 8.3 ± 2.3, *P* < 0.01; 8AA-length, viral epitopes 7.7 ± 2.1, random array 3.0 ± 0.9, *P* < 0.1; and 9AA-length, viral epitopes 4.8 ± 1.1, random array 1.0 ± 0.7, *P* < 0.05. The numbers of matching human proteins for AAs derived from HPV16L1 epitopes were lower than for AAs derived from HBV and RSV epitopes. For example, the numbers of shared AA sequences at the 8AA level were as follows: HPV16L1, 2; HBV sets 1–3, 5 to 16; RSV sets 1–2, 6 and 12; and Random array sets 1–4, 1 to 5. When the length of the AAs was ten or more (where the specificity was high), there was no difference between combined viral epitope sequences versus the Random array sets. (Fig. 1**, **Table 2) (The mean common AA-sequence number ± SEM: 10AA-length, viral epitopes 1.0 ± 0.63, random array 0.3 ± 0.3, *P* = 0.31). All human protein epitopes identical to viral epitopes or Random array sets were listed in Table S10.

Lastly, we conducted gene ontology analysis using human protein epitopes sharing 5 to 11 AA-sequences with HPV16L1, HBV (sets 1–3), and RSV (sets 1–2) epitopes, and four sets of random array sequences. We found that there was no statistically significant enriching gene ontology term in the human proteins sharing with any three viral epitope groups or Random array sets.

## Discussion

Most antibodies have been shown to recognize the three-dimensional structure of epitopes rather than linear AA-sequences of epitopes. Here, when the full-length AA-sequences of a microbial epitopes are found to be identical to those of host protein epitopes, merely having molecular homology at the AA-sequence level could often be insufficient for anti-microbial antibodies to cross-react with host proteins, due to the spatial relationship of the epitope within the entire host protein molecule. In addition, for anti-microbial cross-reactive antibodies to cause cell/tissue damage, the target host epitopes must be on the cell surface for antibody binding. Thus, although Kanduc “discovered” intracellular host protein epitopes possessing molecular mimicries with microbial epitopes, these epitopes cannot be recognized by cross-reactive antibodies, leading to host cell damage.

Theoretically, when an in silico computational analysis shows that the full-length AA-sequences of microbial epitopes are identical to those of human protein epitopes (i.e., molecular mimicry), the microbial proteins have the potential to induce cross-reactive autoantibody production following microbial infections or vaccinations. However, this computational approach has a limitation: Kanduc’s in silico analysis was based on linear AA-sequence databases of protein epitopes, without information on conformational epitopes (such as crystal structures or 3D structure prediction). Thus, antibody binding to the three-dimensional structure cannot be assessed by Kanduc’s methodology. To test clinically relevant molecular mimicry between microbial epitopes and host epitopes, the following immunological assays should be conducted to examine whether anti-microbial antibodies really bind to the host epitopes: an enzyme-linked immunosorbent assay (ELISA) and/or Western blotting with or without adsorption of microbial and host epitopes; cell-based antibody binding assay using cells expressing the target host epitopes; and immunofluorescent antibody binding assay on tissue sections expressing the target host epitopes. These binding assays should be further required to include functional assays using cell lines and animals to determine whether the cross-reacting antibodies can cause antibody-mediated cell and organ damage through molecular mimicry. In our study, using the same approach as Kanduc’s group [16], we examined the antibody epitopes of HPV16L1 and found that none of the entire AA-sequences of the 22 linear epitopes (Table 1) were identical to any AA-sequences of human protein epitopes. Our result was consistent with findings by Kanduc’s group [11, 16], who showed that there were no human protein epitopes identical to the HPV16L1 epitopes, although Kanduc’s group has never emphasized these crucial findings.

Kanduc reported that approximately 2,000 pentapeptides and hexapeptides derived from HPV proteins were screened against all 36,103 human proteins to determine whether each human protein contained at least one identical sequence, and that the corresponding matching rates were 42.5% and 4.7%, respectively. Since peptides are composed of 20 types of amino acids, Kanduc presented the theoretical probability of a given pentapeptide or hexapeptide matching another pentapeptide or hexapeptide as 1/20^5^ and 1/20⁶, respectively. Kanduc then concluded that the observed matching rates were higher than expected based on these probabilities [15]. However, Kanduc’s approach is scientifically flawed because it neither takes into account the length of human proteins nor compares the results with the matching rates obtained using random sequences. In fact, in our analysis, 248 pentapeptides extracted from random amino acid sequences matched 578–972 different human proteins, and 237 hexapeptides matched 28–60 different human proteins **(**Table 2**)**.

We found that HPV16L1 had fewer partial AA-sequence similarities than HBV and RSV epitopes. Although such “partial” molecular mimicry is irrelevant to the induction of cross-reactive autoantibodies, our results demonstrated that HPV16L1 epitopes were not unique in having a certain number of partial AA-sequence similarities with human protein epitopes, compared with other viral protein epitopes.

We also demonstrated that viral protein epitopes (HPV16L1, HBV, and RSV) had more partial AA-sequence similarities with human protein epitopes than randomly generated AA sequences (i.e., Random array sets 1–4) (Fig. 1). Despite the evolutionary distance, humans and viruses may share specific sequence features that are biologically relevant for protein formation. On the other hand, for HPV16L1 epitope sequences, concordance with human proteins was equivalent to random sequences for AA-sequences of eight or more. Thus, human proteins did not have a highly specific match with the HPV16 L1 epitope sequences, although identical AA-sequences were commonly found at shorter AA-length levels (Fig. 1).

Epidemiologically, HPV vaccinations have been demonstrated not to increase the incidence of immune-mediated diseases or any diseases [21, 22]. Experimentally, no researcher, including Kanduc's group, has ever demonstrated cross-reactivity between the HPV L1 protein and human tissues/proteins by ELISA or other methods using anti-HPV sera, anti-HPV L1 antibodies, or autoantibodies [10, 23]. Clinically, no studies have shown antibody deposition in the organs, increased autoantibody production, or efficacy of immunotherapies in alleged patients with HANS. It is now widely accepted in the scientific community that there is no point in suggesting the risk of autoimmune diseases by demonstrating the sharing of short peptide sequences by in silico analysis without supporting epidemiological data or experimental evidence [24–27].

Kanduc et al. performed computational analyses on severe acute respiratory syndrome coronavirus 2 (SARS-CoV-2) vaccines, using the same computational approach in their HPV vaccine studies, and reported that the SARS-CoV-2 vaccines could be dangerous due to the presence of 5–7 AA-sequences shared between SARS-CoV-2 and human proteins [28–31]. Kanduc’s group also reported that vaccines against meningococcal B [32], diphtheria toxin [33], RSV [34], and HBV [11, 35] could pose risks due to the presence of 5–7 shared AA-sequences with human proteins; none of these studies, however, experimentally tested whether vaccine-induced antibodies could cross-react with the plausible microbial epitopes or damage/kill the potential target human cell types/organs. As described above, the mere observation that certain AA-sequences of microorganisms have similarities to human proteins should not be used as a scientific basis for asserting vaccine risks.

This study has a limitation. This is an in silico analysis restricted to available databases and does not account for conformational epitopes. In fact, the affinity in the three-dimensional structure cannot be assessed in this current model. However, this limitation also applies to Kanduc's research, and by performing the same analysis as her, we highlighted this limitation in her work.

In conclusion, we demonstrated that the identical AA-sequences to full-length HPV16L1 epitopes were not present among the human protein epitopes. We also demonstrated that HPV16L1 epitopes had fewer partial AA-sequence similarities than HBV and RSV epitopes. Therefore, it is incorrect to assert the risk of HPV vaccinations based on common AA-sequences in the HPV vaccines and human protein epitopes.

## Supplementary Information

Below is the link to the electronic supplementary material.

Supplementary file1 (XLSX 39 kb)

Supplementary file2 (XLSX 131 kb)

## Declarations

### Registry and the Registration No. of the study/trial

N/A

### Animal Studies

N/A
